# Contemporary Multimodality Imaging for Diagnosis and Management of Fabry Cardiomyopathy

**DOI:** 10.3390/jcm13164771

**Published:** 2024-08-14

**Authors:** Simrat Kaur, Jaideep Singh Bhalla, Angelika L. Erwin, Wael Jaber, Tom Kai Ming Wang

**Affiliations:** 1 Section of Cardiovascular Imaging, Department of Cardiovascular Medicine, Sydell and Arnold Miller, Heart, Vascular and Thoracic Institute, Cleveland Clinic Foundation, Cleveland, OH 44195, USA; kaurs4@ccf.org (S.K.); jaberw@ccf.org (W.J.); 2 Department of Internal Medicine, Cleveland Clinic Foundation, Cleveland, OH 44106, USA; bhallaj@ccf.org; 3 Department of Medical Genetics and Genomics, Cleveland Clinic Foundation, Cleveland, OH 44195, USA; erwina@ccf.org

**Keywords:** Fabry disease, infiltrative cardiomyopathies, cardiac MRI

## Abstract

Fabry disease (FD) is an X-linked lysosomal storage disorder which leads to the accumulation of globotriaosylceramide (Gb3) in various organs, including the heart. FD can be subdivided into classic disease resulting from negligible residual enzyme activity and a milder, atypical phenotype with later onset and less severe clinical presentation. The use of multimodality cardiac imaging including echocardiography, cardiac magnetic resonance and nuclear imaging is important for the diagnostic and prognostic evaluation in these patients. There are gaps in the literature regarding the comprehensive description of cardiac findings of FD and its evaluation by multimodality imaging. In this review, we describe the contemporary practices and roles of multimodality cardiac imaging in individuals affected with Fabry disease.

## 1. Introduction

Fabry disease (FD) is one of the most commonly diagnosed lysosomal storage disorder, with the reported incidence ranging from approximately 1:17,000 to 1:117,000. The clinical manifestations of FD are caused by a deficiency of the α-galactosidase A (α-Gal A) enzyme due to pathogenic variants in the *GLA* gene [1]. This deficiency of enzyme α-Gal A leads to the accumulation of globotriaosylceramide (Gb3) and its derivatives. The condition is inherited in an X-linked manner and while males are usually more severely affected, females frequently also present with symptoms [2]. In classic FD, males typically become symptomatic in childhood with often unspecific complaints such as episodes of severe pain in hands and feet. Chronic renal insufficiency resulting in end-stage renal disease and cardiovascular manifestations occur in young adulthood and can lead to debilitating complications and death at a young age. These males with classic FD usually have very little to absent residual enzyme activity. Patients with higher residual enzyme activity develop atypical or late-onset FD. Age of onset as well as phenotypic manifestations are very variable and—depending on the causative *GLA* variant—a single organ system can be affected. Phenotypic variability is most pronounced in affected females, who can occasionally be as severely affected as men or remain asymptomatic throughout their lives, especially if they carry a *GLA* variant that causes atypical disease [3]. Furthermore, while some states in the US have included FD on their newborn screen panels, there is often significant diagnostic delay, especially in atypical or late-onset cases that may present later in life [4]. It is therefore of utmost importance that clinicians maintain a high degree of suspicion for notable presenting signs and symptoms to ensure a timely diagnosis and allow for early treatment initiation to slow down disease progression and prevent long-term complications. FD affects multiple organ systems and need a multispecialty approach with a list of extracardiac manifestations, as summarized in Table 1. We here present a review of the cardiovascular manifestations of FD as well as the utilization of multimodality imaging in managing them, as these remain a significant cause of morbidity and mortality in this patient cohort.

## 2. Cardiac Manifestations of FD

Cardiovascular mortality is the leading cause of death in both classical and cardiac variants of FD [5,6]. The presence of certain cardiac findings should alert clinicians to the presence of underlying FD even in the absence of a suggestive family history or classic extracardiac symptoms.

### 2.1. Left Ventricular and Septal Hypertrophy

Left ventricular hypertrophy (LVH) with/without diastolic dysfunction in the absence of an alternate diagnosis should raise the clinical suspicion for FD [7]. Pieroni et al. have described a concise algorithm to work up unexplained LVH in suspected FD patients, emphasizing a low threshold for confirmatory genetic and enzymatic testing [8]. Heart failure is a significant morbidity in some patients with FD, with a prospective study estimating the prevalence of symptomatic heart failure to be 41% in their cohort of FD patients [9]. Heart failure in FD is thought predominantly to be due to impaired diastolic filling with LVH. On the other hand, a reduction in ejection fraction is uncommon, and possibly underreported in FD, with Wu et al. reporting only 6.8% of patients with an EF < 55% [10].

### 2.2. Cardiac Arrhythmias

Gb3 infiltration of the conduction system of the heart can give rise to a multitude of cardiac arrhythmias. A screening electrocardiogram (ECG) can have findings of LVH, short PR interval, repolarization abnormalities and varying degrees of conduction abnormalities. An increase in the QRS voltages may be seen in these patients, which may not solely be a result of LVH. The biochemical basis for this, as well as a short PR interval (and other associated findings suggestive of accelerated AV conduction) may be explained by facilitated electrical transmission by Gb3, especially in the early stages of the disease and in the absence of a concomitant accessory pathway [11]. In the advanced stages of the disease, as the impairment of the electrical system and fibrosis of these myocyte predominates, a greater prevalence of conduction delays and bradyarrhythmias are observed [11,12]. A systematic review with Baig et al. attempted to evaluate the burden of ventricular arrhythmias (VA) and sudden cardiac death (SCD) in FD patients [13]. They reported the SCD prevalence to be 62%, a major cause of mortality in this cohort. Males, especially over the age of 40 years, had a higher risk of VA/SCD, late gadolinium enhancement on cardiac MRI with the presence of LVH, and a history of non-sustained ventricular tachycardia.

### 2.3. Valvulopathy

Furthermore, valvulopathy can occur due to valvular leaflet thickening and regurgitation, most commonly affecting the mitral and aortic valves [14]. Dilation of the aortic root and ascending aorta can occur due to the fibrotic expansion of the tunica media and associated aortic stiffness [14]. A retrospective review of three European centers with 106 FD patients estimated the incidence of dilatation at the level of sinus of Valsalva and ascending aorta up to 32.7% and 9.6%, respectively [15]. Peter et al. also reported an increased prevalence of aortopathy in individuals with pre-existing cardiac involvement and irrespective of treatment with ERT [16]. Interestingly, the prevalence of aortic dissection remains low with our review yielding a singular case which occurred in the context of an acute COVID-19 infection in a young male who had stopped ERT 2 months prior to presentation [17].

### 2.4. Microvascular Ischemia

Progressive glycosphingolipid accumulation can also present as coronary microvascular dysfunction due to coronary vasculopathy. Acute coronary syndrome is an important consideration in patients with chest pain and merits a similar initial workup as for patients without FD. Angina is a frequent presentation of coronary artery disease in FD patients, which could be a result of epicardial coronary artery occlusion or microvascular ischemia due to glycosphingolipid accumulation in the endothelium [18].

## 3. Cardiac Evaluations and Monitoring

### 3.1. Electrocardiographic Monitoring

**Figure 1:**
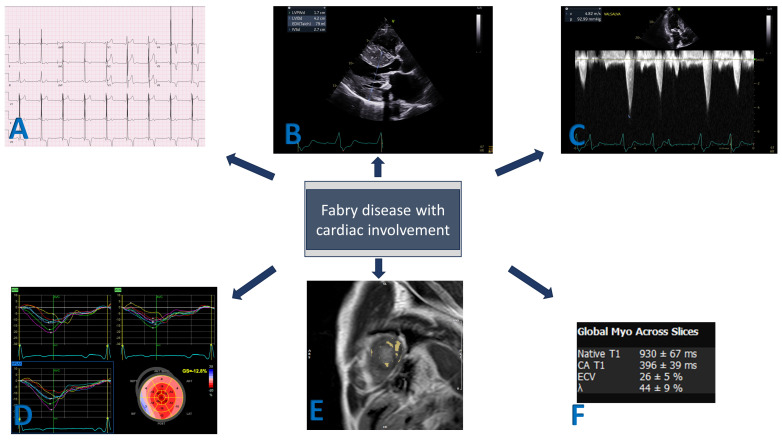
Imaging findings of Fabry-related cardiomyopathy. (**A**) Left ventricular hypertrophy by EKG. (**B**) Left ventricular hypertrophy by echocardiogram. (**C**) Left ventricular outflow obstruction with gradient as observed by echocardiography. (**D**) Global longitudinal strain with reduced strain in the basal inferior segments. (**E**) Cardiac MRI showing late gadolinium enhancement (LGE) in basal inferior and lateral segments. (**F**) T1 mapping with low T1 time and normal extracellular volume.

The spectrum of rhythm abnormalities in FD can extend from the subtle classic electrocardiogram (ECG) changes to varying degrees of conduction blocks and fatal arrhythmias. Bundle branch blocks, pseudo-Q waves, left ventricular hypertrophy, short PR interval and non-specific ST-T wave changes particularly in inferolateral leads due to progressive fibrosis are some of the well-described ECG changes [19] (Figure 1). Therefore, ambulatory rhythm/Holter monitoring is recommended to screen patients who would benefit from cardiac implantable electrical devices (CIEDs) [20].

Holter monitoring is used to establish a baseline and for serial surveillance of arrhythmias, though normal results do not preclude their absence and may indicate the need for an extended duration of rhythm evaluation. Weidemann et al. followed 16 patients with advanced cardiac FD using implantable loop recorders and found 15 events of clinically relevant rhythm disturbances requiring a change in existing management [21]. This further highlights the need for close rhythm monitoring in patients who have features suggestive of progressive myocardial fibrosis.

### 3.2. Echocardiography

Transthoracic echocardiography is the first-line imaging modality for the evaluation of cardiac involvement in these patients (Figure 1). The pattern of LVH is most commonly concentric, but apical or interventricular septal predominant hypertrophy can also be seen. The latter can result in left ventricular outflow tract (LVOT) obstruction and needs differentiation from other causes of hypertrophy, such as hypertrophic cardiomyopathy, hypertensive heart disease and other infiltrative cardiomyopathies, including amyloidosis or Danon disease [22].

Echocardiography can evaluate strain pattern mapping, which has been described as an early predictor of myocardial involvement in FD [23,24]. In patients with histological evidence of glycolipid accumulation, Pieroni et al. were able to demonstrate reduced myocardial contraction as well as relaxation velocities even prior to the development of LVH, raising the possibility of a pre-clinical diagnosis. This may certainly indicate a need for closer monitoring in affected patients, but it is unclear whether this cohort would benefit from Fabry-specific treatment once advanced fibrosis has ensued. The measurements for tissue Doppler velocities, contraction and relaxation time, and the E/e′ ratios need to be evaluated in the appropriate clinical context to determine if they are pathological, since attempts to standardize these data have been very challenging and of poor utility to recommend changes in management for these patients [5]. These patients should undergo a comprehensive echocardiographic evaluation for the assessment of LV volumes and ejection fraction, RV size/function analysis as well as the conventional parameters to assess diastology, in accordance with the 2016 ASE guidelines for the evaluation of LV diastolic function on echocardiography [25]. It is important to note that while the presence of the following thresholds may strongly indicate the presence of diastolic dysfunction on an echocardiogram, these parameters have not specifically been validated in the FD cohort and, therefore, must be interpreted with caution: E/e′ > 14, e′ septal < 7 cm/s/lateral < 10 cm/s, Left atrial volume indexi > 34, tricuspid regurgitation >2.8 m/s, E/A ratio, isovolumetric relaxation time, as well as left atrial reservoir strain <18%.

Speckle tracking echocardiography, by virtue of providing an estimation of the regional function across all segments of the myocardium, adds a crucial advantage over the conventional two-dimensional echocardiography and has the advantage of being quicker than Doppler-based strain imaging [26,27]. Basal lateral and posterior segments of the LV are predominantly involved in the disease process, and speckle tracking can provide a surrogate marker for the evaluation of delayed enhancement on CMR, which in turn would provide an assessment of the degree of myocardial fibrosis and has potential treatment implications. Evaluations of the systolic longitudinal systolic strain and diastolic strain rate are the preferred indices for the determination of diastolic dysfunction in FD and are independent of the presence of an increased LV wall thickness or mass [28]. Speckle tracking also allows for the assessment of left atrial (LA) hemodynamics, using parameters such as the strain rate and LA volumes across different phases of the cardiac cycles. This not only provides an additional criterion for the early diagnosis of Fabry cardiomyopathy but could also have prognostic implications pertaining to the development of atrial arrhythmias and may be reversible with enzyme replacement therapy [29].

Both conventional and speckle tracking echocardiography can also assist in the evaluation of any pericardial effusions, the RVSP and the valvular apparatus in FD patients. Gb3 accumulation within the valves as well as the chordae can result in varying degrees of stenosis and regurgitation, respectively. These in turn can further contribute to the progression of heart failure and arrhythmogenic complications. Likely owing to a greater hemodynamic stress, the aortic and mitral valves are disproportionately affected in the disease process, with some burden likely resulting from secondary processes as a result of the aortic and left ventricular enlargement, respectively [30]. Notably, pericardial effusion has been rarely reported as a direct consequence of FD due to globotriaosylsphingosine accumulation and an uncertain response to enzyme replacement therapy [31].

### 3.3. Cardiac Magnetic Resonance Imaging

Whilst echocardiography findings are typically non-specific, cardiac magnetic resonance imaging (CMR) can identify more specific features to aid the diagnosis and surveillance of FD (Table 2). The 2020 AHA/ACC guidelines for HCM recommend CMR imaging for patients with LVH when there is a suspicion of alternative diagnosis [32]. The 2020 expert consensus on FD also recommends imaging with CMR for an accurate assessment of LV geometry, size and mass with gadolinium contrast when possible. The application of multiparametric CMR also provides valuable insights into the potential mechanisms of LVH and fibrosis as well as sex-related differences.

**Figure 2:**
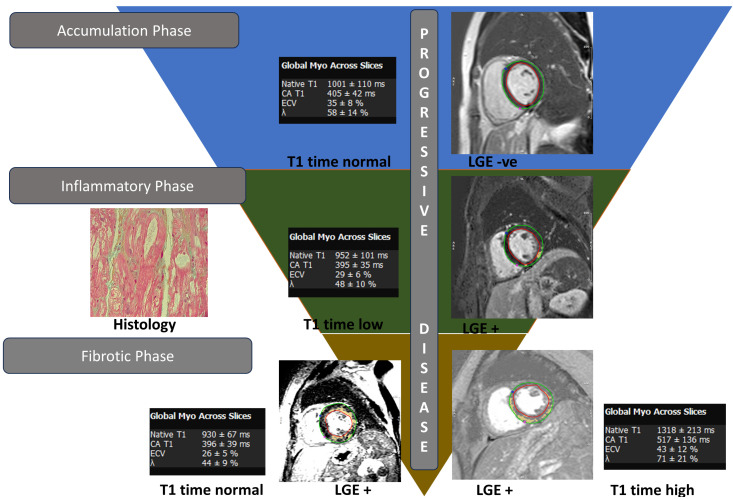
Cardiac MRI findings in a patient with Fabry disease and its progression through various stages.

The unique ability of CMR is to accurately evaluate ventricular dimensions, volumes and mass thickness. CMR has a higher spatial resolution than echocardiography and can provide highly reproducible measurements that are less operator-dependent [33]. In addition, CMR can provide tissue characterization by utilizing gadolinium contrast agents as well as proton relaxation that would give T1, T2 and T2* relaxation times (Figure 1). Patients with Fabry cardiomyopathy typically show late gadolinium enhancement (LGE) in the basal inferolateral LV wall in a mid-myocardial distribution (Figure 2). Less commonly, mid-myocardial LGE can also be seen in the basal anteroseptum in the presence of septal hypertrophy, and apical segments in the presence of apical hypertrophy. The differential distribution of LGE can be attributed to fibrosis, and although HCM, myocarditis, sarcoidosis and Chagas disease may have similar findings, the mechanism is different. For example, intramyocardial ischemia in hypertrophied areas leads to fibrosis, while subclinical inflammation, which is more prominent in the mid-myocardial and basal–mid-inferolateral wall is a feature of Fabry cardiomyopathy [34,35,36]. In some instances, LGE may be observed before LVH develops in women with FD. This finding is particularly important given the elevated risk of worse cardiac outcomes (i.e., arrhythmias) with LGE in these patients. Additional studies investigating noninvasive cardiac markers such as myocardial strain, which are less load-dependent, have also been reported, potentially helping with early diagnosis [37,38]. Given the increase in risk, there is a suggestion of primary prevention with implantable cardioverter defibrillators in patients with excessive LGE, although this practice has not been included in the consensus statement [13,39].

T1 mapping and extracellular volume fraction (ECV) is another critical component of CMR FD evaluation. Low native T1 relaxation times are sensitive for Fabry cardiomyopathy and often correlate with abnormal LV-GLS using feature-tracking CMR [40]. Nordin et al. proposed stages of myocardial phenotype evolution in FD: (1) accumulation phase, starting in childhood, which results in the progressive lowering of T1 time with a normal LGE due to the subclinical accumulation of glycosphingolipids in the myocardium; (2) inflammatory phase, when myocyte hypertrophy ensues, which presents as LGE typically in the basal inferolateral segments along with low T1 time; (3) and fibrosis, which is the final stage that occurs after persistent LVH and subsequent thinning with LGE, particularly in basal inferolateral segment [41,42]. This delineation is important as extensive fibrosis has been associated with an increased risk of arrhythmias as well as suboptimal response to enzyme replacement therapy (ERT) [43,44].

The relationship of T1 with LVH is sex-specific, with T1 time falling markedly in men when compared with women. The low values of T1 time are particularly found in the interventricular septum owing to the increased myocardial lipid levels [45]. In women, T1 time returns to normal levels after LVH develops but in men, T1 time increases significantly with LVH. The degree of LVH is also more profound in men as well as the increased prevalence of LGE. This differential finding can be explained by sex-related differences with myocyte hypertrophy as a response to storage, leading to increased sarcomeric protein expression and an increase in T1 time, while in women, there is a balance of LVH and storage of sphingolipids, subsequently diluting the T1 time. Several studies have also reported the appearance of LGE in women before LVH occurs [46,47,48].

ECV is calculated by a comparison of pre- and post-contrast T1 relaxation times of the myocardium with the blood pool, adjusted for hematocrit:

Myocardial ECV = (1 − hematocrit) × (ΔROI myocardium/ΔROI blood)

In healthy controls, ECV is known to be higher in women, which also extends to FD with ECV levels being lower in men compared to women [49,50].

CMR is also utilized for longitudinal follow-up for the screening of LVH and monitoring of response to Fabry-specific treatment [51,52]. ECV levels can be elevated in patients with amyloidosis due to extracellular fibril deposition; however, elevation in its absence can be a marker for myocardial fibrosis. FD leads to intracellular glycosphingolipid deposition with normal ECV levels; however, advanced disease with fibrosis can increase levels of ECV [53].

T2-weighted sequences can help identify areas of myocardial edema which can be inflammatory or non-inflammatory and, unlike T1 mapping, follow a linear progression of disease. Fabry patients can have elevated T2 values in areas depicting LGE, as well as globally, which can be a potential biomarker for risk stratification and monitoring therapies [50,53]. Chronic elevation in T2 time in areas of LGE has been associated with clinical worsening and chronic troponin leak [54].

### 3.4. Nuclear Scintigraphy and Positron Emission Tomography

FD can cause the intramural narrowing of coronary arteries due to smooth muscle proliferation, endothelial dysfunction due to Gb3 storage, and microvascular remodeling, which in advanced cases, can lead to replacement fibrosis [55,56]. This gives rise to microvascular dysfunction that is often independent of LVH and, in advanced cases, can result in coronary artery pruning because of decreased vessel diameter from smooth muscle hypertrophy [57].

The evaluation of angina in cases of FD can be performed with SPECT 99 mTc Sestamibi or Positron Emission Tomography (PET) scans, which show a global reduction in coronary flow in the absence of epicardial coronary vessels, suggestive of microvascular dysfunction [55]. Tomberli and co-authors assessed hyperemic myocardial blood flow values using ammonia-PET in patients with FD and found that the flows were markedly blunted independent of gender or any other cardiac involvement [57]. They also found a worsening impairment in myocardial blood flow in patients with LVH in contrast to those without. This is an important observation suggesting coronary microvascular dysfunction as an important early manifestation of FD independent of LVH, which would be critical in starting early targeted therapy.

Recent studies have also investigated the use of hybrid cardiac 18F-Flourodeoxyglucose (FDG) PET-CMR imaging, that can detect myocardial inflammation in areas of 18F-FDG uptake with the corresponding increased signal intensity of T2 short inversion recovery imaging [36,58]. Another study examining the impact of hybrid 18F-FDG PET/CMR imaging in females with FD found focal (lateral) 18F-FDG uptake as a sign of early disease, which correlated with impaired longitudinal strain on echocardiography. The majority of patients in this study exhibited no evidence of LGE and negative T2-STIR on CMR (91.6%; *n* = 24) irrespective of focal or diffuse 18-F-FDG uptake, which demonstrates heterogenous sensitivity of CMR in early disease [59].

Patients with FD often have symptomatic peripheral and autonomic neuropathy due to the deposition of glycosphingolipids in neural cells. The 123I-metaiodobenzylguanidine (MIBG) SPECT scan is a noninvasive scan that measures ganglionic norepinephrine uptake assessing the adrenergic innervation of the heart. In a recent study by Imbriacco et al., a reduction in the uptake of 123I-MIBG was seen in the inferolateral LV wall in the absence of fibrosis on concomitant CMR, suggesting abnormalities in myocardial adrenergic function in patients with FD [60]. A possible explanation of this finding is the predominance of parasympathetic neurons in the inferior wall compared with the anteroseptal wall, resulting in the early depletion of the sympathetic neurons [61]. When compared with echocardiography, longitudinal systolic strain derangement is an earlier manifestation of cardiac involvement in contrast to myocardial neuronal damage, which again demonstrates the utility of a multimodality imaging approach when managing FD patients [62].

## 4. Management

### 4.1. Treatment of Fabry Disease

**Figure 3:**
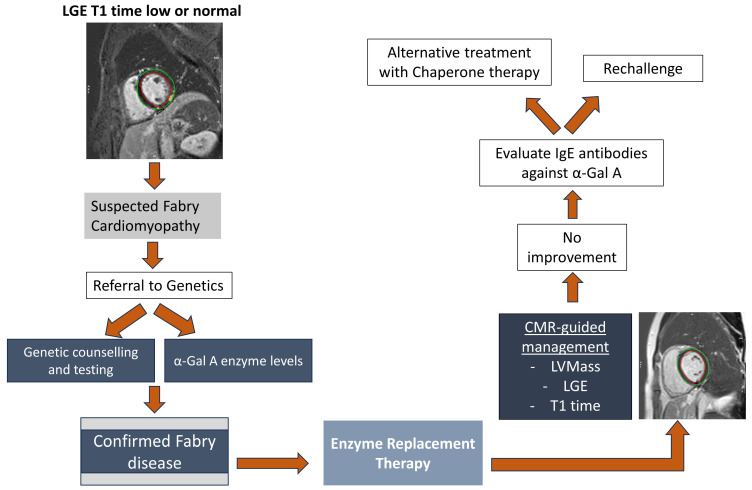
Diagnostic and treatment algorithm in a patient with suspected Fabry cardiomyopathy.

Given the multisystemic nature of FD, a multidisciplinary team including specialists in cardiology, nephrology, genetics, and neurology is required to provide comprehensive care directed at control of disease-related symptoms and prevention of disease progression [63]. Current FD-specific therapies include enzyme replacement therapy (ERT) and chaperone therapy, the latter of which is available for patients with select *GLA* variants (Figure 3).

#### 4.1.1. Enzyme Replacement Therapy

ERT has changed the disease trajectory in patients with FD and has been shown to reduce the rates of severe clinical events such as renal failure, cardiac events, stroke, and death [7]. FDA-approved ERT products include agalsidase beta (Fabrazyme^®^, Sanofi US, Bridgewater, NJ, USA) and pegunigalsidase alfa (Elfabrio^®^, Chiesi USA, Cary, NC, USA). Recommendations for monitoring of disease manifestations and treatment initiation have been published for adult as well as pediatric patients. Generally, treatment is recommended as soon as FD-related subclinical manifestations seen on laboratory, histological or focused imaging tests are detected, even in the absence of clinical manifestations, which in males is commonly at the time of diagnosis [64,65]. ERT is administered intravenously every two weeks. Improvement of gastrointestinal and neurological disease manifestations have been reported [14,66,67]. Studies have also shown delay in cardiovascular disease progression along with the regression of LVH in select cases [68].

#### 4.1.2. Chaperone Therapy

Chaperone therapy with migalastat (Galafold^®^, Amicus Therapeutics, Philadelphia, PA, USA) is an important alternative treatment option in patients with FD. Chaperones act by binding to the active site of the α-Gal A enzyme, promoting its proper folding and trafficking to lysosomes. Migalastat is administered orally but patients need to have an amenable *GLA* variant to be eligible for this medication [69]. Treatment with migalastat has been reported to be associated with a reduction in the left ventricular mass [70].

### 4.2. Management of Cardiac Symptoms

Patients with heart failure are managed according to the 2022 AHA/ACC/HFSA and 2023 focused update on 2021 ESC guidelines, save for some restrictions [71,72]. As mentioned previously, caution should be implemented while using nodal blocking agents. The surveillance of hyperkalemia in patients with compromised renal function and receiving guideline-directed medical therapy is necessary. Cardiac resynchronization therapy with pacemaker implantation should be considered in patient with systolic dysfunction (ejection fraction < 50%) along with widened QRS (>120 ms). Patients with obstructive cardiomyopathy who have resting or exertional symptoms resistant to medical therapy should be considered for septal reduction therapies (alcohol septal ablation or septal myectomy). AV nodal blocking agents should be used with caution in patients without extrinsic pacemaker therapy. Disopyramide also needs renal dose adjustment in FD patients with renal insufficiency [14].

Standard antianginal therapies may be considered to manage anginal chest pain. In patients with coronary artery disease, medical treatment includes antiplatelet agents, statins and antianginals as well as revascularization with drug-eluting stents for focal obstructive lesions. Caution should be implemented while using negative inotropes such as beta blockers, calcium channel blockers and ivabradine, which can increase the risk of bradyarrhythmias.

As per the expert consensus, maintenance of rhythm control is recommended in patients having atrial fibrillation [14]. Screening for arrhythmias should be performed by 48 h Holter monitoring. All patients with atrial fibrillation or flutter should receive anticoagulation irrespective of CHADS2VAS2c score. The chronic use of amiodarone has been shown to induce phospholipidosis, which can lead to progressive renal insufficiency and decrease the effect of ERT [73,74]. The importance of the timely initiation of anticoagulation for atrial fibrillation or flutter and CIED implantation for conduction blocks/ventricular events to prevent morbidity and mortality in this cohort cannot be overstated.

Dual-chamber pacemaker implantation can be considered in symptomatic patients with chronotropic incompetence [14]. The prevention of sudden cardiac death is more nuanced due to lack of data. While ICD implantation is recommended in patients with a history of cardiac arrest or with hemodynamically unstable VT, it can also be considered in patients with extensive LVH and advanced fibrosis, although these decisions should be tailored to each individual patient based on risk factors.

## 5. Conclusions

Fabry disease is subject to several challenges for earlier diagnosis and can often present with cardiac involvement leading to significant morbidity and mortality. The utilization of noninvasive imaging modalities in addition to genetic and enzymatic testing and managing these patients in a multidisciplinary approach are critical. Increasing awareness and early initiation of therapies can help alter the trajectory of this illness.

## Figures and Tables

**Table 1:** Summary of common extracardiac clinical manifestations of Fabry disease classified by organ system.

**Neurological**	
1. Peripheral Nervous system	
(A) Sensory	Acroparesthesias, pain crises/Fabry crises, varying degrees of sensory loss (small-fiber neuropathy/altered temperature perception: hypo/anhidrosis)
(B) Autonomic	Orthostatic hypotension and syncope due to vasomotor involvement
2. Central Nervous System	Most common: Intracranial vasculopathy manifesting as small/large vessel strokes; Periventicular white matter lesions and verterbrobasilar dolichoectasias, and common imaging findings Less common: Neuropsychiatric involvement with cognitive dysfunction may be observed
Ocular	Cornea verticillata Retinal vasculopathy Premature cataracts
Auditory/Vestibular	Hearing loss Tinnitus Vertigo
**Dermatological**	Characteristic lesions: Angiokeratomas
**Gastrointestinal**	Dysregulation of gut motility due to enteric/autonomic neuropathy or GI smooth muscle involvement: leads to early satiety, vomiting/nausea, constipation or diarrhea and weight loss
**Renal**	Accelerated development of proteinuric chronic kidney disease: due to glomerular (both endothelial/podocytes), tubular and interstitial sclerosis

**Table 2:** Characteristics of various multimodality. Multimodality imaging for the evaluation of cardiac involvement in Fabry’s disease.

Technique	Role in Disease Management	Advantages of Technique	Disadvantages of Technique
**Conventional echocardiography**	Identification of cardiac involvement in asymptomatic patients such as LVH, reduced tissue Doppler velocity. Longitudinal monitoring of patients to response to ERT.	Rapid, easily accessible, inexpensive. Early detection of diastolic dysfunction can assist in pre-clinical diagnosis (prior to development of LVH).	Operator-dependent; non-specific.
**Speckle tracking echocardiography**	Allows for the early evaluation of cardiac involvement in patients with FD, thereby having implications for prompt ERT initiation.	Easy access and quick to perform. Act as a surrogate marker for LGE on cMRI.	Operator-dependent; non-specific. Lack of standardized measurements across different machines.
**Cardiac MRI**	Confirmatory testing after identification of LVH on echocardiography to allow for the precise quantification of LV mass (can also be used for longitudinal disease monitoring). Evaluation of low T1 intensity in suspected patients may allow for the early diagnosis of cardiac involvement.	High spatial resolution. Accurate and highly reproducible measurements of LV geometry. Standardized measurements, allowing for establishing reference values. Allows for disease characterization in cases of unexplained LVH.	Not readily accessible. Expensive. Limitations in those without compatible metallic implants.
**Nuclear scintigraphy/PET**	Assessment of microvascular dysfunction. Evaluation of areas of myocardial inflammation with hybrid imaging.	Noninvasive measurement of coronary blood flow.	Non-specific. Expensive. Not readily available.

## Data Availability

No new data were created or analyzed in this study. Data sharing is not applicable to this article.
